# Agro-waste extracted cellulose supported silver phosphate nanostructures as a green photocatalyst for improved photodegradation of RhB dye and industrial fertilizer effluents†

**DOI:** 10.1039/d0na00181c

**Published:** 2020-06-17

**Authors:** Neha Tavker, Umesh K. Gaur, Manu Sharma

**Affiliations:** School of Nano Sciences, Central University of Gujarat Sector 30 Gandhinagar 382030 India manu.sharma@cug.ac.in; Department of Physics, National Institute of Technology Jalandhar Punjab 144011 India

## Abstract

The efficiency and reusability of photocatalysts are the dominant factors for their pragmatic use. The visible light induced semiconductor silver phosphate is a superior photocatalyst effective under visible light but its stability is still an undiscussed issue. To overcome this stability issue in this present manuscript, eco-friendly agro-waste extracted cellulose supported silver phosphate nanostructures have been designed for the first time through a simple chemical process. At first, silver phosphate nanostructures were synthesized by the co-precipitation method. Then, different weights of cellulose were added to the silver nitrate solution to form cellulose supported silver phosphate nanostructures. The photodegradation efficiency for each weight ratio was examined in which the photocatalyst Ag-8 nanostructures showed a high rate (0.024 min^−1^) for degradation of Rhodamine B (RhB) using a low intensity tungsten bulb. Real sample analysis has also been carried out using this photocatalyst for the degradation of industrial fertilizer effluents. The degradation rate of all the nanostructures was found to be high in comparison to pristine silver phosphate as well as the extracted bare cellulose. The photocatalytic activity is enhanced because of the participation of cellulose as a support which makes an interface for silver phosphate and assists it in delaying the charge recombination period under visible light. To understand the photochemical reaction of electrons and holes, scavenger studies were also performed.

## Abstract 2

Re-utilization of waste for fabricating photocatalyst to treat polluted water.

## Graphical abstract

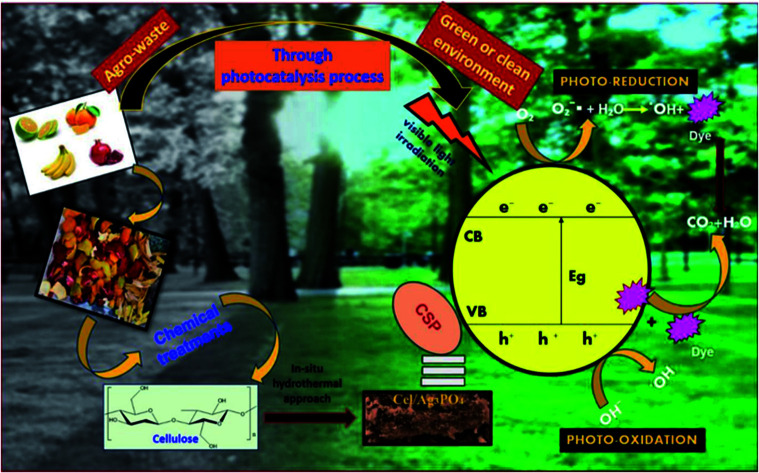

## 1. Introduction

As industries have been a major source for the release of hazardous chemicals or effluents such as drugs, dyes, fertilizers, pesticides, heavy metals, and nitro compounds and their derivatives, since then the polluted water has become a major global issue for the modern society.^1^ Contamination found today is mainly due to the inaccurate methods of waste disposal whether it may be medical, industrial or agricultural waste. Therefore there is an urgent need for researchers to find new techniques for complete removal of the pollutants or to make them non-toxic to discharge them into the groundwater.^2^ According to a survey, during manufacturing processes, 10–15% of environmental water gets polluted with dyes. Some dyes are highly stable and very difficult to degrade which can cause health hazards to humans, flora and fauna.^3^ Over the last couple of decades, research on the multidisciplinary field of semiconductor photocatalysts has grown tremendously due to their potential to solve two major issues related to shortage of renewable energy resources and that of the environmental deterioration and their various applications like bactericidal coatings, photocatalysis, self-cleaning, new generation solar cells, environmental remediation, and hydrogen production and sensing.^4–7^ However, for achieving success in this field, it is desirable to develop high performance semiconducting materials having a wide light response range, efficient charge separation ability and sufficient energy of charges which can photodegrade dyes and other toxic molecules.^8,9^The most reported ones are TiO_2_ and ZnO, which urges us to focus on generating novel active visible light semiconductor photocatalysts.

There are many reports on synthesis of nanostructures *via* thermal decomposition, co-precipitation and hydrothermal methods.^10,11^ PbS, NiO, CuS, Cu_2_S, Mn_3_O_4_ and MgO have been synthesized by Niasari’s group.^11–14^ Nanodimensional pores of zeolite-Y encapsulated with Mn(ii), Co(ii), Ni(ii), Cu(ii), and Zn(ii) complexes have also been synthesized using Schiff's base.^15^ Among all other photoactive compounds, n-type semiconducting silver orthophosphate with a band gap of 2.36 eV (ref. 16) is a promising visible light photocatalyst due to its superior capability for photooxidation by producing oxygen by water splitting in the presence of visible light.^17^ Also it has been proven that organic dyes can be degraded more efficiently in its presence.^18^ Moreover, to achieve high photodegradation efficiency, quick recombination of charge carriers should be avoided as it reduces the quantum efficiency of the photocatalyst. In order to enhance the photodegradation efficiency and overcome the stability issue Ag_3_PO_4_-based composites were synthesized by researchers which include SnO_2_/Ag_3_PO_4_,^19^TiO_2_/Ag_3_PO_4_,^20^ AgX/Ag_3_PO_4_,^21^ Fe_3_O_4_/Ag_3_PO_4_,^22^ Ag/Ag_3_PO_4_ (ref. 23) and so on. Currently, carbon material/Ag_3_PO_4_ nanocomposites (carbon quantum dots (CQDs)/Ag_3_PO_4_,^24^ graphene/Ag_3_PO_4_ (ref. 25) and CNT/Ag_3_PO_4_ (ref. 26)) are being used to improve the photodegradation efficiency and stability of Ag_3_PO_4._ Due to their exceptional structure and charge transfer ability, CNTs are known to decrease the electron–hole recombination which in turn increases the photocatalytic activity under visible light. However, non-renewable resources have also been used for the synthesis of CNTs and this remains a challenge for green synthesis. Ternary composites with expensive supports are reported for the fabrication of versatile photocatalysts. So, there arises a need for fabricating a less toxic and renewable material as a substitute for CNTs through an economical approach.

The polymeric cellulose, which is available excessively, has the potential to resolve the issues related to material biodegradability, renewability, toxicity, and cost and it may also pose as a better replacement for CNTs. Cellulose is commonly found in lignocellulosic biomass,^27^ derived from agricultural leftovers and it may add one more feature in the green synthesis. Using this agro based waste by refining it with chemical treatments is one of the green routes and cost-effective routes for producing cellulose. Of all the bio-templates, cellulose is the most renewable biomass which gives a skeletal structure and a possible support for visible light photocatalysts. Moreover, its structure has plenty of electron rich hydroxyl groups^28^ which assist the interaction with the photocatalyst due to the electrostatic interaction. It can be obtained from a variety of renewable bioresources such as wood, cotton, bacterial cellulose, agricultural crops, and agricultural cellulosic wastes.^29^ Wood and cotton have been considered as the most important source of cellulosic fibres; however, concerns for the environment and shrinking of forest caused by the increased use of wood resources led to growing curiosity in the exploitation of non-wooden cellulosic materials.^30–32^ Cellulose until now has been used in the pharmaceutical industry,^33^ food industry,^34^ medical industry,^35^ paper industry^36^ and cosmetics.^37^ In pharmaceutics it is used in osmotic drug delivery systems,^38^ extended and controlled release matrices,^39^ extended and delayed release coated dosage forms, bio adhesives, mucoadhesives,^40^ granules and tablets as binders.^41^ Thus, the research on cellulose supported photocatalysts is still inadequate. Hence, cellulose hybrid nanocomposites may be one of the suitable photocatalysts not only from a green chemistry point of view but also due to their high efficiency and better stability.

The main purpose of the present work is to develop an advanced cellulose/Ag_3_PO_4_ visible light photocatalyst by improving the stability of Ag_3_PO_4._ This would include isolating the cellulose from waste fruit rinds by using a support, thereby reducing the cost of the material. To the best of our knowledge, a cellulose/Ag_3_PO_4_ composite with the cellulose isolated from fruit rind waste having efficient visible light photocatalytic performance has not yet been reported. Here we report a cellulose supported Ag_3_PO_4_ photocatalyst with increased photocatalytic activity towards Rhodamine B (RhB) dye and industrial effluents under visible light by improving the reaction rate. This work provides advanced insights and basis of understanding photocatalytic mechanisms of hybrid photocatalytic systems. Also, it would provide an alternative use of disposed domestic and commercial waste.

## 2. Materials and methods

### 2.1 Chemicals

Sodium chlorite, potassium hydroxide, sodium hydroxide, glacial acetic acid, silver nitrate, rhodamine B, and *p*-benzoquinone were obtained from Sigma Aldrich. Sodium phosphate dibasic, EDTA, 2-propanol and sulphuric acid were obtained from SRL, India.

### 2.2 Methodology

#### 2.2.1 Isolation of cellulose

100 g peels, each of different seasonal fruits (sweet lime, bananas, pomegranates and oranges) were taken and fruit bran was prepared from these waste peels by following steps like washing and drying. Then the dried sample was ground and sieved using a 60 mm mesh sieve. All the further steps were followed as described in our previous reported method where 15 g of yellow colored bran was used to extract cellulose with chemo-mechanical treatments.^42,43^ The first step of isolation involved alkali hydrolysis with 5% w/v KOH with stirring for 12–14 h. The suspension was centrifuged and neutralised at each step. The second step involved bleaching with 1% NaClO_2_ at pH-5 at 70 °C. This step de-lignified the lignin present in bran used as the source. The last step was acid hydrolysis with 1% H_2_SO_4_ at 80 °C for 1 h. The steps were repeated accordingly as per need. The final suspension was centrifuged, washed, neutralized and stored at 4 °C. The suspension was dried by lyophilisation and was utilized further for characterization.^44^ The extracted cellulose was coded as Cel (steps followed are shown schematically in Fig. S1†).

#### 2.2.2 Synthesis of cellulose supported silver phosphate nanostructures

Further, we synthesized nano-sized silver phosphate and its cellulose supported silver phosphate nanostructures by varying the weights of cellulose along with silver phosphate. Silver phosphate (SP) particles were synthesised *via* the co-precipitation method. For the synthesis of silver phosphate nanostructures, 0.06 M of silver nitrate and 0.02 M of sodium phosphate were taken in 50 ml of double distilled water each. 0.2 g of CTAB (cetyltrimethylammonium bromide) was added to control the size of silver phosphate during the reaction which aided in the formation of nano-sized particles. This sample was coded as Ag-0. Cellulose supported silver phosphate nanostructures were prepared by an *in situ* approach by adding 0.2 g, 0.5 g and 0.8 g of cellulose in silver nitrate solution followed by the dropwise addition of disodium hydrogen phosphate to the reaction mixture during the reaction along with CTAB. All the as prepared samples were coded as Ag-2, Ag-5 and Ag-8 for cellulose supported silver phosphate nanostructures. For the *ex situ* method a 1 : 1 weight ratio of cellulose and silver phosphate was added in 100 ml of deionized water and kept in a sonicator for 1 h. The products were collected by repeated washing and centrifugation to remove the surfactant. This sample was coded as Ag-1 for *ex situ* cellulose supported silver phosphate nanostructures.

### 2.3 Characterization methods

Powder X-ray diffraction patterns (PXRD) of all the samples were obtained using a Panalytical's Xpert Pro with CuKα mono-chromatized incident radiation of wavelength 0.1540 nm operated at a scanning speed of 10° min^−1^. Measurements were carried out using an incidence detector at a glancing angle of 2°, in the 2*θ* range from 10° to 60°. The % reflectance and band gap of all the photocatalysts were calculated through Diffuse Reflectance Spectroscopy (DRS) (Jasco 670). Fourier Transform Infrared Spectroscopy (FTIR, Perkin Elmer Sp65) was performed in the range 400–4000 cm^−1^ to assign the functional group of the samples. For surface topography of bran and cellulose suspensions, Bruker's Multimode 8 Atomic Force Microscope (AFM) was used under tapping mode. Samples used in AFM imaging were prepared on a 1 cm × 1 cm square glass cover slip and dried for 1 h in a vacuum oven. Surface morphology and elemental analysis was carried out on a JEOL JSM 6390LV field emission scanning electron microscope (FESEM). Dry solid powder was used for FESEM analysis and the samples were fixed on carbon tape followed by gold coating. A Jeol/JEM 2100 was used to record electron micrographs, selected area diffraction patterns and HRTEM images. A minute amount of sample was dispersed in isopropanol and 1 drop of this solution was loaded onto a carbon coated copper grid and allowed to dry before analyzing. Lifetime measurements of the photocatalyst were performed using a JOBIN VYON Fluorocube with an excitation wavelength of 390 nm. An Omicron ESCA, Germany was used for obtaining XPS spectra. An aluminium anode was used for samples with an approximate energy of 1486.7 eV. The angle between the source and analyzer was 85°. Monochromatic X-rays were used whose resolution was confirmed by an FWHM of 0.6 eV. The Brunauer–Emmett–Teller (BET) specific surface area, pore size distribution and pore diameter were obtained using adsorption–desorption nitrogen isotherms at a liquid nitrogen temperature of 77 °C using Quanta chrome Nova Win station equipment. Fluorescence lifetime measurements were performed using a JOBIN VYON Fluorocube system with an excitation wavelength of 390 nm. For these measurements 20 mg of samples were dispersed in de-ionized water and analyzed. A digital pH meter was used to measure the pH value of samples. The photocatalytic activities of all the catalysts were examined by measuring the absorbance of the filtered mixture using a UV-Vis spectrophotometer (Shimadzu UV probe 2.52). Fertilizer effluents collected from Kalol, Gandhinagar were analyzed using a Waters Xevo G2S Qtof LCMS.

### 2.4 Photocatalytic measurements

For the photocatalytic degradation of the RhB dye, 25 mg of the catalyst was added individually in 25 ml of 10 μM of RhB dye solution and irradiated under visible light with a tungsten lamp (200 W) until it degraded completely. The light intensity calculated theoretically was found to be 707 W m^−2^ with a distance of 15 cm from the light source. The wavelength of the lamp was found to be 301 nm. After adding the photocatalyst the dye solution was stirred in the dark for 30 min to ensure adsorption–desorption equilibrium. Then Ag-0 and cellulose were tested for photodegradation efficiencies. All the other nanostructures were also used to photodegrade the RhB dye until its optimal point.

Further, to understand the roles of photogenerated electrons and holes during the photochemical reaction, different scavengers such as isopropyl alcohol, benzoquinone and EDTA were used as hydroxyl, superoxide and hole radical scavengers. The photodegradation efficiency of the finest catalyst was analyzed by conducting the experiments individually with 1 mM of each scavenger. Recyclability and photostability of this catalyst were also checked for 5 cycles under visible light irradiation for 60 min. All these experiments of photocatalysis were performed in triplicate and the kinetic parameters were obtained using an Origin 8.5 by linear regression analysis.

For the real sample analysis, industrial fertilizer effluents were collected from a fertilizer industry located at Kalol, Gandhinagar and analyzed individually as industrial fertilizer waste and a mixture of fertilizer waste and the standard dye sample. These sets of samples were examined by using the best photocatalyst in the presence of visible light. The photocatalytic studies were performed for 3 sets:

(1) RhB dye + catalyst (Cat) as a standard sample

(2) Fertilizer effluent (FE) + catalyst (Cat) as a real sample

(3) RhB dye + fertilizer effluent (FE) + catalyst (Cat) as a real sample

All 3 sets of samples were kept under a visible light source in the presence of a catalyst. The blank experiment was also performed to check the photodegradation efficiency of the catalyst.

## 3. Results and discussion

### 3.1 XRD analysis

Powder XRD patterns of the fruit bran and isolated cellulose are shown in Fig. S2† as reported in our previous work also.^45^ A considerable enhancement was observed in the crystallinity of cellulose as compared to fruit bran. The diffraction peaks of cellulose can be indexed to (110) (200) and (004) which are in accordance with cellulose-I with a monoclinic structure.^46^ As described by Zhang and Lynd,^47^ cellulose possess a crystalline structure due to van der Waals forces and H-bonding making an interface in it. The XRD results were in accordance with the reports of recent years which confirmed the phase purity of isolated cellulose.^48–50^ However, a reasonable amount of the cellulose structure is disordered and often referred to as amorphous. The crystallinity Index (CI) was measured from the ratio of the height of the intense crystalline peak (*I*_002_ − *I*_AM_) to total intensity (*I*_002_) after deduction of the background signal measured without cellulose. The CI for fruit bran was found to be 46% which increased to 74% in isolated cellulose after chemical treatments of hydrolysis and bleaching.

**Fig. 1:**
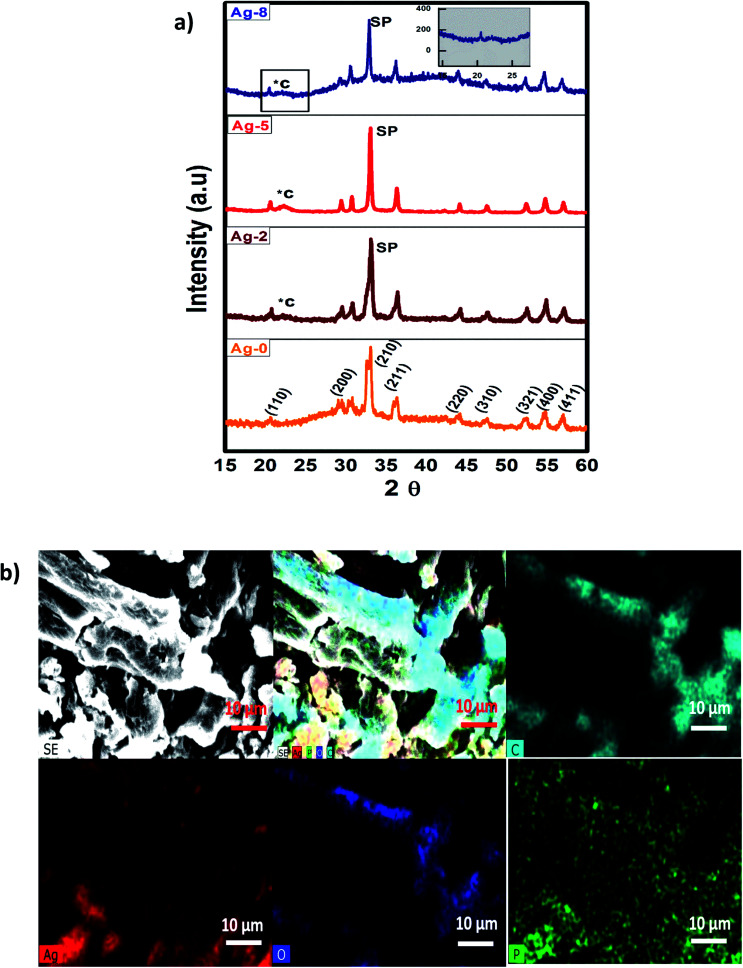
(a) X-ray diffraction patterns of cellulose supported Ag_3_PO_4_ nanostructures [Ag-0–Ag-8]. (b) SEM-EDX mapping images of cellulose supported silver phosphate nanostructures (Ag-8).

The XRD patterns of samples Ag-0, Ag-2, Ag-5 and Ag-8 are shown in Fig. 1a which confirm the formation of body centred cubic silver phosphate. The diffraction peaks of Ag-0 correspond to the planes (110), (200), (210), (211), (220), (321), (400) and (411) respectively which are in accordance with JCPDS file no. 84-0512.^51^ All the samples exhibit the formation of a pure phase without any impurities. The peaks of cellulose in the nanocomposites are depicted by *C and peaks of silver phosphate as SP. Fig. 1b shows the elemental map of the Ag-8 sample, where all the elements have been represented by different colours. It is obvious that silver phosphate nanoparticles are dispersed on the cellulose surface. The crystallite size is the smallest, most likely a single crystal in a powder form. The crystallite size (*D*) was found using the Scherrers formula (0.9*λ*/*β* cos *θ*) *via* an Origin 8.5. The average crystallite size for Ag-0, Ag-2, Ag-5 and Ag-8 was found to be 1.31, 9.66, 15.81, and 6.39 nm respectively. The larger size of Ag-8 is due to attachment of the cellulose crystal.

### 3.2 FTIR spectroscopy

Fig. S3a in the ESI† displays the FTIR spectra of bran and isolated cellulose. The broad peak seen in the range of 3400–3420 cm^−1^ is attributed to O–H stretching, 2920–2922 cm^−1^ depicts the =C–H group, 1400–1600 cm^−1^ is attributed to C=C stretching, 1050–1060 cm^−1^ is due to C–O stretching, and 400–700 cm^−1^ is attributed to C–H bending. After the alkali treatment a sharp peak at 1550 cm^−1^ appears due to removal of lignin and hemicellulose.^52,53^ Fig. S3b† shows the FTIR spectra of Ag-0 to Ag-8 *in situ* and *ex situ* cellulose supported Ag_3_PO_4_ nanostructures. The two strong bands at 1000 cm^−1^ and 544 cm^−1^ are attributed to molecular vibration of phosphate ions (P–O) which confirms the metal phosphate bonding of the catalyst.

### 3.3 Diffuse reflectance spectroscopy

The change in the optical properties was analyzed by DRS measurements (Fig. S4†). The band gaps of all the catalysts were calculated using the Kubelka–Munk equation and plotted as a function of absorption co-efficient *versus* band gap energy of all the samples. As cellulose has insulating properties it has a band gap > 4.0 eV which is not clearly seen in the visible region. So, the band gap of Ag-0 which was found to be 2.48 eV started decreasing (from 2.485 to 2.464 eV) as the amount of cellulose was increased. The band potentials for Ag-0 was calculated theoretically which showed 0.21 eV for *E*_CB_ and 2.69 eV for *E*_VB_.

### 3.4 Thermal analysis

Thermal stability was analyzed by thermogravimetric analysis (TGA) for FB, Cel, Ag-0 and Ag-8. Fig. S5† shows TGA and DTA (Differential Thermal Analysis) curves for the above mentioned samples. Heat was continuously given up to 860 °C which caused the weight loss. Bleaching and alkali treatments increases stability of the materials due to the presence of contents like lignin, hemicelluloses, pectin *etc.* They have lower decomposition temperatures as compared to cellulose supported nanostructures. The rise in temperature of Ag-0 and Ag-8 is due to the presence of the ash content. The purpose of the pre-treatment in the isolation process is to eliminate a certain amount of lignin or minimize the quantity of the wax, hemicellulose, and oils that cover the fibrillary outer surface of the wall of the cell. The depolymerisation of the native structure of cellulose defibrillates the outer cellulose micro fibrils and exposes short length crystallites that can be found due to the alkali treatment. The bleaching step is also crucial as it removes the cementing material completely from the fibre.^54^ Acid hydrolysis is performed at last which disseminates into the province of lignocellulosic biomass and easily splits the nanowhiskers.^55^

### 3.5 Atomic force microscopy

The change in surface properties of FB and Cel has been examined by AFM studies after isolation and purification steps. Fig. S6† shows AFM images of FB (Fig. S6a and b†) as well as cellulose (Fig. S6c and d†) which was used as a support. As it can be seen in Fig. S5a,† roughness is more in the sample because of agglomerated particles. When cellulose is isolated from fruit bran the particle shows less roughness as shown in Fig. S6c.† The average particle size of cellulose through AFM was found to be ∼48.2 nm.

### 3.6 FESEM-EDAX and HRTEM analysis

The morphology of FB, Cel, and Ag-0 to Ag-8 nanostructures was analyzed by FESEM. The FESEM image of fruit bran (FB) (Fig. S7a†) shows irregular shaped particles. Cellulose was found to have of a rod shaped morphology with an aspect ratio of ∼2.6 (Fig. S7b†). The FESEM image and EDAX pattern of Cel are shown in Fig. S7c.† The EDAX spectrum also confirms the presence of carbon and oxygen in the cellulose with a higher amount of oxygen.

**Fig. 2:**
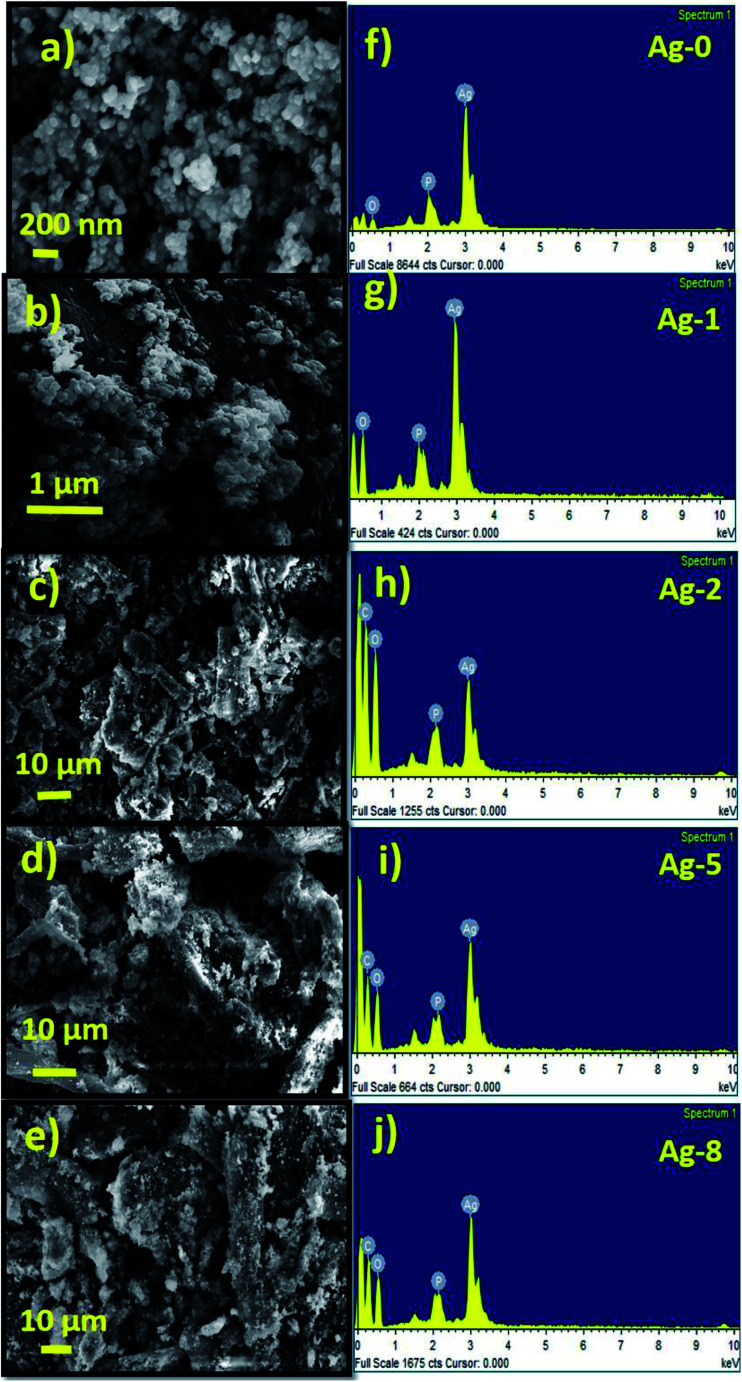
FESEM micrographs of pure silver phosphate [Ag-0 (a)] and cellulose supported silver phosphate nanostructures [Ag-1 (b)], [Ag-2 (c)] [Ag-5 (d)] and [Ag-8 (e)] and their respective EDAX patterns (f–j).

The FESEM image of pure silver phosphate nanoparticles (Ag-0) shows close to a spherical type morphology with an average size of 50 nm as shown below (Fig. 2a). Fig. 2f shows the EDAX spectra of Ag-0. In Fig. 2b, *ex situ* cellulose supported silver phosphate nanostructures (Ag-1) are shown. All the *in situ* cellulose supported silver phosphate nanostructures (Ag-2, Ag-5 and Ag-8) exhibit spherical shape particles over the surface of the rod shaped cellulose support (Fig. 2c–e). Silver phosphate nanoparticles present on the rod shaped cellulose have an average particle size of 25 nm. The EDAX pattern of all the cellulose supported nanostructures also assures the existence of the elements; mainly carbon, oxygen, phosphorous and silver in the Ag-1, Ag-2, Ag-5, and Ag-8 nanostructures (Fig. 2f–j).

**Fig. 3:**
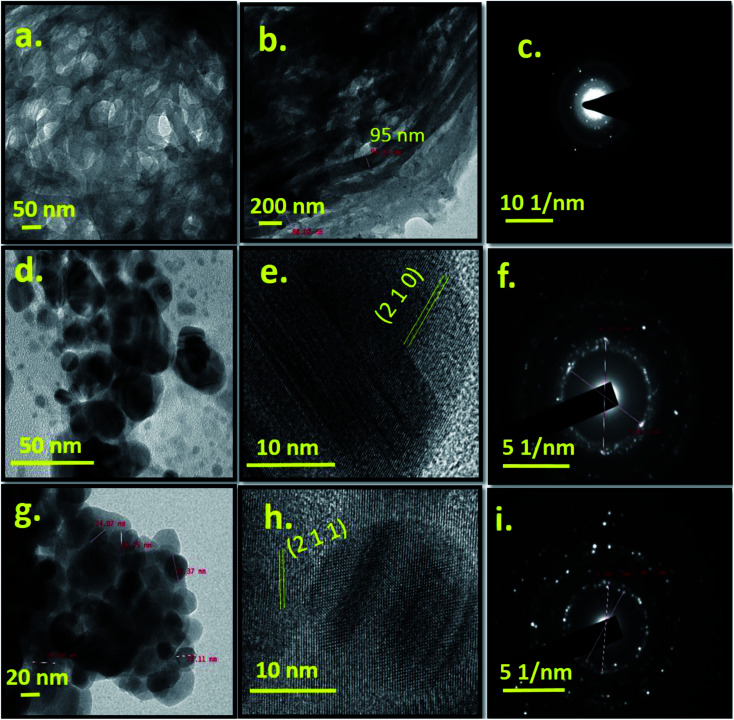
TEM image, and HRTEM and SAED patterns of cellulose (Cel) [a–c], silver phosphate (Ag-0) [d–f] and cellulose supported silver phosphate nanostructures (Ag-8) [g–i].

Fig. 3 shows TEM and HRTEM images of Cel, Ag-0 and Ag-8 along with their respective SAED patterns. Cellulose particles are micron sized and also undergoe electron collision. So, the morphology of particles is not very clear from TEM images at this scale bar. But the HRTEM image shows a fibrous type structure. Fig. 3b shows the size of cellulose which ranges between 88 and 95 nm in diameter. Fig. 3c shows diffused rings which confirm the polycrystalline nature of the cellulose. The TEM image of the best photocatalyst (Ag-8) shows a spherical particle with a size range of ∼25–30 nm (Fig. 3d). Pure silver phosphate nanoparticles confirmed the specific planes along with the highly polycrystalline nature through HRTEM and SAED patterns with the (200) and (211) planes respectively (Fig. 3e and f). Fig. 3g shows spherical particles ∼25–30 nm on the surface of cellulose. The HRTEM image shows the (211) plane of the silver phosphate and Fig. 3i shows the SAED pattern due to the polycrystalline nature of the silver phosphate in the Ag-8 nanostructure.

### 3.7 X-ray photoelectron spectroscopy

**Fig. 4:**
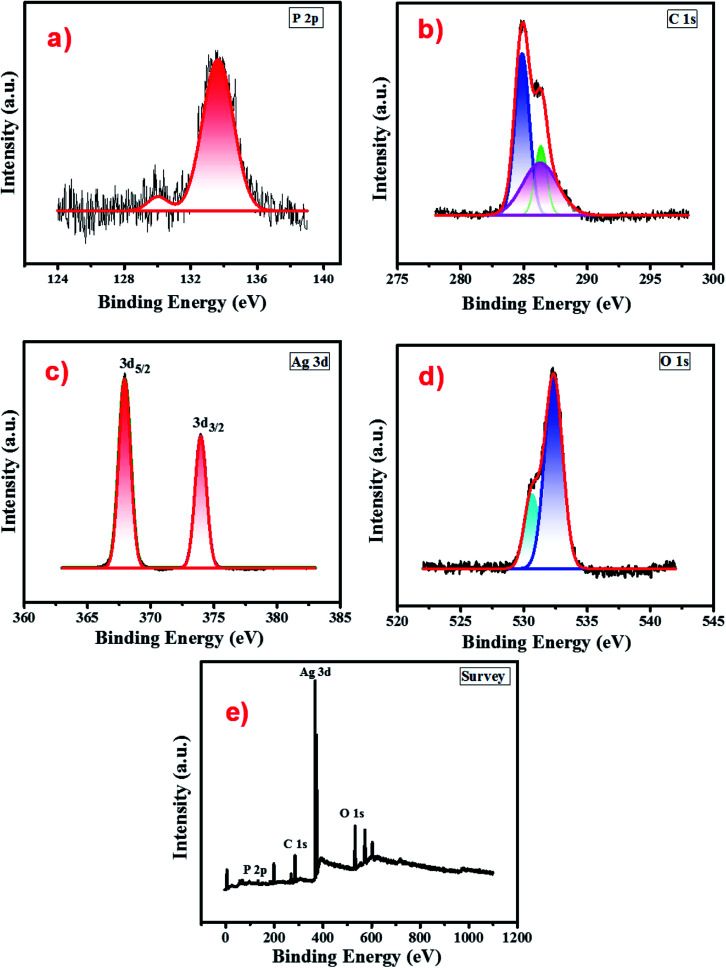
XPS spectra of the Ag-8 photocatalyst (a) P 2p (b) C 1s (c) Ag 3d (d) O 1s and (e) survey scan.

The surface chemical composition and oxidation state of atomic species present in the cellulose supported Ag_3_PO_4_ nanostructures were analysed by recording the XPS spectrum in the binding energy range 0–1100 eV. Fig. 4 presents the survey scan of the cellulose supported silver phosphate and it shows the peaks related to Ag, P, C and O without other impurity elements. The high-resolution spectra of each element *i.e.* Ag 3d, P 2p, C 1s and O 1s respectively, are shown in different panels of Fig. 4. Two peaks in the P 2p spectrum (panel (a)) the smaller one at 130.02 eV and the other larger one at 133.65 are associated with the P–C bond due to the cellulose interaction^56^ and the presence of phosphorus in the phosphate group of Ag_3_PO_4_ (ref. 57). As compared to the reported data for pure Ag_3_PO_4,_ an additional small peak (130.02 eV) attributed to the interaction of cellulose with Ag_3_PO_4_ nanostructures can be observed. The XPS spectra of C 1s (panel (b)) depict three peaks located at 284.8 eV, 286.2 and 286.4 eV. The peak at 284.8 eV is typical of the C–H bond in cellulose^58^ while peaks at 286.2 and 286.4 eV are associated with C–O bonding and ensure the cellulose–Ag_3_PO_4_ interaction.^59^ Panel (c) describes the XPS spectrum of the Ag 3d core level and reveals two peaks, one at 367.9 (3d_5/2_) and the other at 373.9 eV (3d_3/2_) with a spin orbit separation of 6 eV which confirms the presence of silver in the Ag^+^ state.^60^ The O 1s spectrum (panel (d)) shows a sharp peak associated with one small shoulder peak at a lower binding energy and de-convoluted into two peaks by fitting. The smaller peak at a binding energy 530.56 eV may be due to the non-bridging (P=O) oxygen atoms. From the absence of a peak at a binding energy 528.6 eV it can be concluded that there is no oxidation of Ag to Ag_2_O.^61^ Another peak observed at a binding energy 532.34 eV can be assigned to P–O–Ag bonding in Ag_3_PO_4_.^62^

### 3.8 Time-resolved photoluminescence spectroscopy and BET surface analysis

**Fig. 5:**
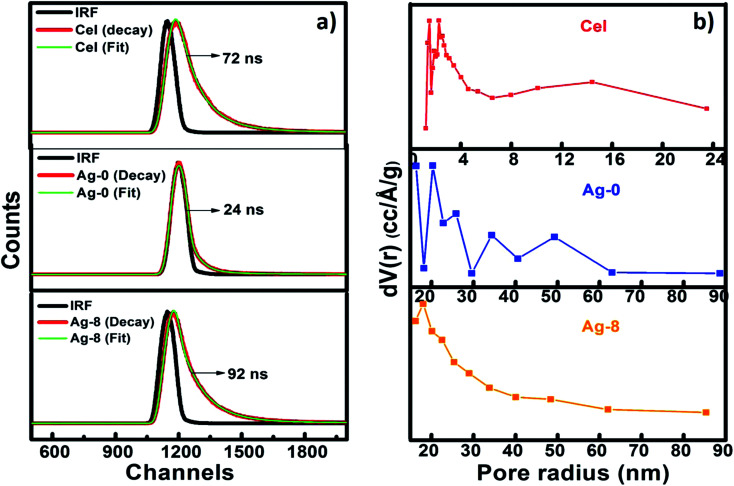
(a) Fluorescence lifetime analysis of cellulose (Cel), silver phosphate (Ag-0) and cellulose supported silver phosphate nanostructures (Ag-8). (b) BJH pore size distribution of Cel, Ag-0 and Ag-8 nanostructures.

Time-resolved photoluminescence (TRPL) is a fast electronic deactivation process that results in the emission of photons, a process known as fluorescence. The lifetime of a photocatalyst in its lowest excited singlet state usually ranges from a few picoseconds up to nanoseconds. Here, for lifetime measurements, Time-Correlated Single Photon Counting (TCSPC) was used for data acquisition. Fig. 5a shows the time resolved spectra of Cel, Ag-0 and Ag-8. After fitting lifetime data, decay parameters consisting of average lifetime and chi-square values of Cel, Ag-0, and Ag-8 are shown in Table 1. It is evident from the table that the presence of cellulose in the Ag_3_PO_4_ photocatalyst increased the average lifetime from 24 to 92 ns due to increase in the lifespan of electron and holes by development of an interface between Ag_3_PO_4_ and cellulose by delaying the charge recombination period to assist in efficient visible light photocatalysis.

**Table 1:** Time resolved fluorescence data of Cel, Ag-0 and Ag-8 nanostructures

Sample	*B*_1_	*Τ*_1_ (ns)	*B*_2_	*Τ*_2_ (ns)	*B*_3_	*Τ*_3_ (ns)	*Τ*_av_ (ns)	*χ* ^2^
Cel	71.41	1.239	23.54	2.754	5.05	10.49	72	1.180
Ag-0	60.98	0.064	32.89	0.759	6.13	4.17	24	0.900
Ag-8	23.10	0.04	68.85	1.534	8.05	9.242	92	1.288

BET theory aims to explain the physical adsorption of gas molecules on a solid surface and serves as the basis for an important analysis technique for the measurement of the specific surface area of a material. Fig. 5b shows BJH pore size distributions of Cel, Ag-0 and Ag-8 nanostructures as a mesoporous behaviour of materials is observed. The surface area analysed by BET and BJH methods and pore size distributions and the pore volumes of Cel, Ag-0 and Ag-8 photocatalysts are summarized in Table 2. The average pore size of Cel, Ag-0 and Ag-8 was found to be ∼1.6 nm, ∼1.9 nm and ∼1.7 nm respectively. Cellulose isolated from fruit bran showed a BJH surface area of ∼83 m^2^ g^−1^ while that of Ag-0 was found to be ∼22 m^2^ g^−1^. The best catalyst (Ag-8) had the highest surface area of ∼117 m^2^ g^−1^. The improvement in the surface area of Ag-8 is due to the presence of the cellulose support. The pore volume of Ag-8 is also greater (0.168) as compared to that of Ag-0 and Cel. Thus, the time resolved spectra proved the fast decay of Ag-0 and Cel whereas the lifetime of Ag-8 was found to be greater.

**Table 2:** BET and BJH surface areas, the pore volume and the pore size of Cel, Ag-0 and Ag-8 nanostructures

Sample	BET surface area (m^2^ g^−1^)	BJH surface area	Pore volume (cm^3^ g^−1^)	Pore radius (Å)
Cel	49	83	0.136	∼16
Ag-0	29	22	0.025	∼19
Ag-8	204	117	0.168	∼17

### 3.9 Photocatalytic studies

**Fig. 6:**
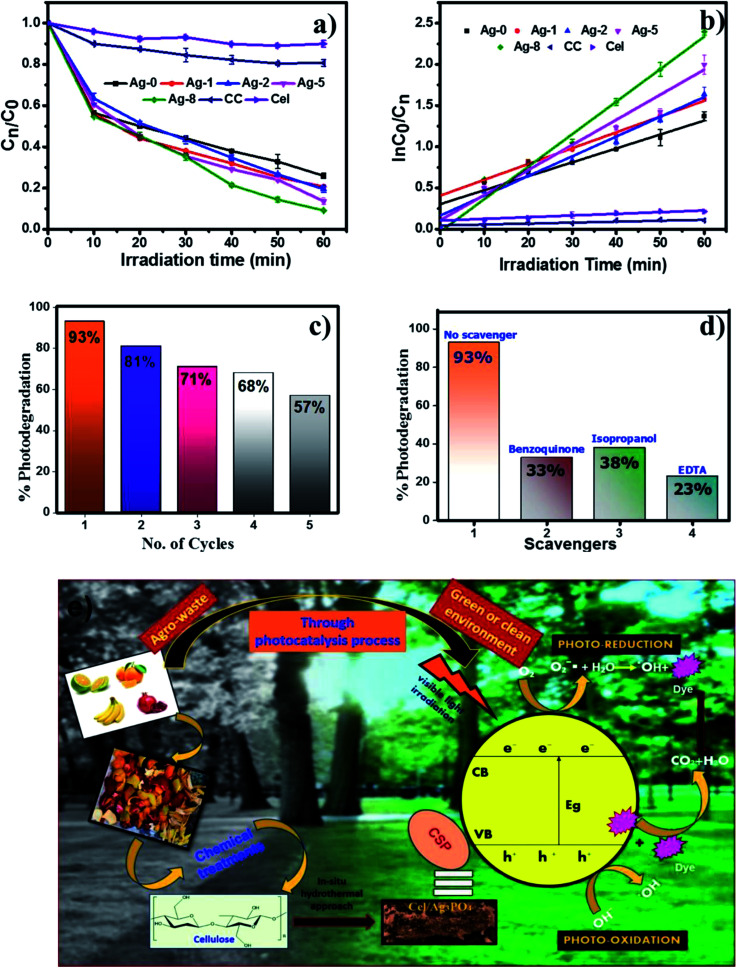
(a and b) Comparative study of kinetic linear simulation of photo degradation of RhB dye using bare Ag_3_PO_4_, cellulose, commercial cellulose and cellulose supported nanostructures. (c) Recyclability tests of the Ag-8 photocatalyst. (d) Scavenger studies for Ag-8. (e) Proposed photodegradation pathway for Ag-8.

To check the photoactivity of all the catalysts, a model RhB dye was used under a visible light source of 200 W. 25 mg catalyst in 25 ml of dye solution was taken to check the photodegradation of RhB. First, the absorption of the catalyst was examined by keeping under dark conditions to maintain the equilibrium of the reaction mixture and later under a light source for 60 min. These experiments were also carried out for determining the % degradation efficiency of all catalysts as shown in Fig. 6. Fig. 6b is the kinetic linear plot for all the photocatalysts which shows the first order reaction kinetics with the improved rate fitted with regression analysis. The catalyst which is loaded on the cellulose remains the same. Only cellulose weights have been varied. So the main factor increasing the rate with a high amount of cellulose is attributed to enhanced charge separation which makes an interface between silver phosphate and cellulose. Ag-8 is the optimal point where the maximum interfaces were developed and the maximum photocatalytic rate was observed. The other higher amounts of cellulose were also used for photocatalysis which showed similar photocatalytic rates, but Ag-8 is the best observed catalyst amongst all.

#### 3.9.1 Photocatalytic stability

Apart from the photodegradation efficiency of the catalysts, stability is also considered one of the important factors. Hence, the recyclability of catalysts was checked for 5 consecutive cycles same as the other photodegradation reaction sets (25 mg catalyst in 25 ml of RhB dye solution). After every cycle, the photocatalysts were separated, washed and dried for further use. The degradation efficiency of the Ag-8 catalyst decreased by only 36% after 5 repetitive cycles. This can be attributed to cellulose in the catalyst being utilized in each photodegradation cycle. The amount of catalyst also gets reduced by weight (∼2–3 mg) at each step in work-up activities which in turn reduces the % degradation efficiency. These results are also supported by scavenger studies and the inhibited photodegradation of RhB is shown as compared to that with a ‘no scavenger system’ (Fig. 6d).

Ag-8 nanostructures showed 96% degradation in 60 min. This result suggested that the photodegradation rate of the Ag-8 catalyst was increased by twice that of Ag-0. Commercial cellulose (CC) was also tested to analyse the photodegradation efficiency for RhB dye. *Ex situ* (Ag-1) and *in situ* samples with a low amount of cellulose [Ag-2 to Ag-5] did not show photocatalysis up to the mark, and hence Ag-8 was chosen as the best photocatalyst. The rate constant value and half-life of the catalysts are tabulated in Table 3. The highest observed rate was 0.024 min^−1^ by using the Ag-8 photocatalyst in which the amount of cellulose is maximum. From Table 3, it is concluded that all the *in situ* samples show high photodegradation rates while *ex situ* samples show lower rates due to improper binding of the support. The bare cellulose and silver phosphate showed lower values which increased after the incorporation of cellulose as a support in all the nanostructures. Based on the band gap measurements and scavenger studies, possible pathways for degradation of RhB dye have been proposed and are shown in Fig. 6e.

**Table 3:** Degradation efficiencies, rates and half-lives of nanocomposites within 60 min presence of visible light

Samples	Rate (min^−1^)	Half life	% error	% degradation
Ag-0	0.0101	69	0.05	73
Ag-1	0.0103	67	0.07	65
Ag-2	0.0104	66	0.08	77
Ag-5	0.015	46	0.07	87
Ag-8	0.024	30	0.07	96
Cel	0.001	693	0.06	20
CC	0.0008	866	0	12

#### 3.9.2 Quenching studies

The effect of scavengers was studied by following the same experimental procedure of photocatalysis. In this study, 1 mM EDTA, benzoquinone and isopropanol each were used for the photodegradation with the Ag-8 catalyst. EDTA was added in the reaction system as a hole scavenger, benzoquinone as a quencher of superoxide and isopropanol as a free radical scavenger during the photocatalysis reaction. In the absence of any scavenger, the catalyst shows very good photocatalytic activity which proves that the reaction intermediates play a crucial role in the reaction along with the visible light source. In Fig. 6d, the inhibited photodegradation of RhB is shown as compared to the photodegradation with a ‘no scavenger system’. The rate constant, half-life and % degradation of all nanostructures [Ag-0 to Ag-8] are shown in Table 3 which indicate a high rate of Ag-8 nanostructures and the maximum photodegradation up to 96% within 60 min whereas commercial cellulose (CC) and extracted cellulose (Cel) just show 12% and 20% photodegradation efficiency in 60 min.

Table 4 shows cellulose based composites used in the literature for photocatalytic degradation of various dyes and drugs. S. Wang and co-workers developed cellulose/Ag@AgCl which degraded Methyl Orange dye within 180 min (ref. 63) using a 500 W xenon source. Nano TiO_2_/cellulose acetate was used in the presence of UV light to degrade Methyl Orange dye within 55 min.^64^ Y. Luo and J. Huang used cellulose with anatase titania with 300 W mercury as the source to degrade Methylene Blue within 35 min.^65^

**Table 4:** Composites of cellulose used in the literature for photocatalytic degradation of pollutants

Composites	Pollutant	Degradation time	Light source	Reference
Cellulose–Ag@Agcl	MO dye	180 min	Visible, 500 W Xe	50
Nano TiO_2_/cellulose acetate	MO dye	55 min	UV light, 8W	51
Anatase titania/cellulose	MB dye	35 min	Mercury lamp, 300W	52
TiO_2_/cellulose	Phenol	140 min	UV light, 6W	53
Nano ZnO/GO/NC	Ciprofloxacin	120 min	Solar light, 100 MW m^−2^	55
ZnO/cellulose nanofibers	RhB dye	24 h	Visible, 500 W tungsten	56

Phenol degradation was also carried out by J. Zeng and team in the presence of UV light using TiO_2_/cellulose.^66^ Ag_3_PO_4_/cellulose was synthesized by Q. Wang and co-workers for photodegradation of RhB but the degradation time was 6.5 h in the presence of sunlight.^67^ The use of nano ZnO/GO/NC has also been reported in the work by T. S. Anirudhan for ciprofloxacin degradation.^68^ A tungsten 500 W visible light source was used to degrade RhB dye with ZnO/cellulose nanofibers with a degradation time 24 h.^69^ Composites with Ag_3_PO_4_ with loading and embedding of various compounds including graphene oxide are shown in Table 5. Q. Wang and co-workers^71^ showed photocatalytic degradation of RhB under sunlight with cellulose hydrogels, whereas our work focuses on isolation of cellulose by reusing waste. Zurui D. and team^72^ showed *in situ* growth of Ag–SnO_2_ quantum dots on Ag_3_PO_4_ for photocatalytic degradation of carbamazepine.

**Table 5:** Composites of silver phosphate reported in the literature with varied light sources

Composites	Pollutant	Light source	Reference
Ag_3_PO_4_/GO	TBBA	35 W Xe	70
Ag_3_PO_4_/cellulose	RhB	Sunlight	71
Ag–SnO_2_ QD on Ag_3_PO_4_	Carbamazepine	Sunlight	72
Graphdiyne/Ag_3_PO_4_	MB	500 W Xe	73
Ag_3_PO_4_/GO/chitosan	RhB, bisphenol-A	210 W Xe arc	74
BiVO_4_/RGO/Ag_3_PO_4_	4-Nitrophenol, RhB	300 W Xe	75
Ag_3_PO_4_/MWCNTs@PANI	RhB	300 W Xe	76
Ag_3_PO_4_/SBF/GO	Phenol, bisphenol-A	35 W Xe	77
Ag_3_PO_4_/GO	MB	350 W Xe	78
Our work	RhB, fertilizer effluent	200 W tungsten	—

Thus, owing to economic and cost constraints, we have developed a cellulose supported silver phosphate photocatalyst *via* a hydrothermal route. Also waste fruit peels were used for extracting cellulose which paved a green route for the synthesis.

### 3.10 Real sample analysis using standard and industrial fertilizer effluents

Although fertilizers are useful for good crop production and in the agriculture field, the release of fertilizer effluents from industrial units directly or indirectly affects groundwater which is considered fatal and toxic for flora, fauna and humans. Wastewater from fertilize plants comprises heavy metals, suspended solids, ammonia, nitrates, and many organics.^79,80^ In the presence of visible light, this type of fertilizer effluent can be treated efficiently by photocatalysts.

**Fig. 7:**
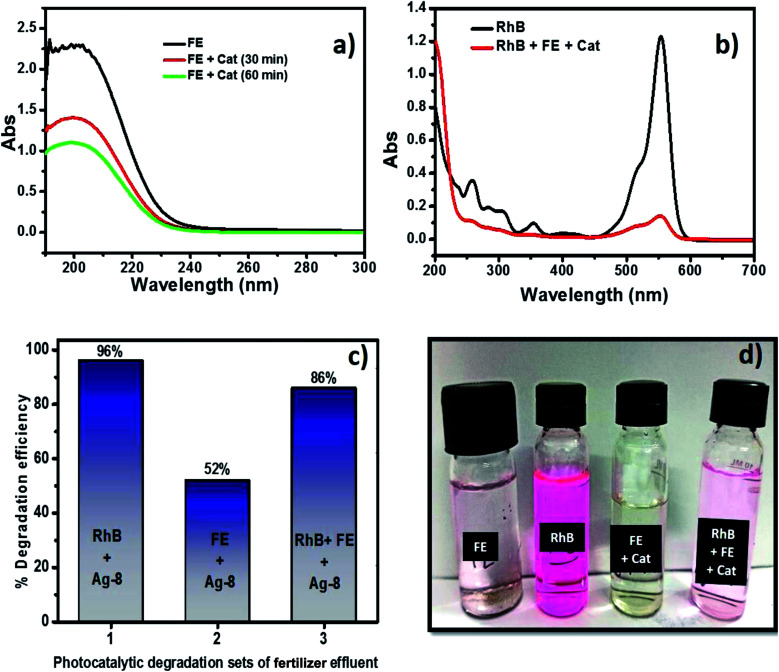
(a) Photodegradation of fertilizer effluents using Ag-8 nanostructures. (b) Photodegradation of the fertilizer effluent and RhB dye with Ag-8 nanostructures. (c) Bar diagram of photocatalytic degradation of different sets. (d) Colour change before and after photodegradation.

**Fig. 8:**
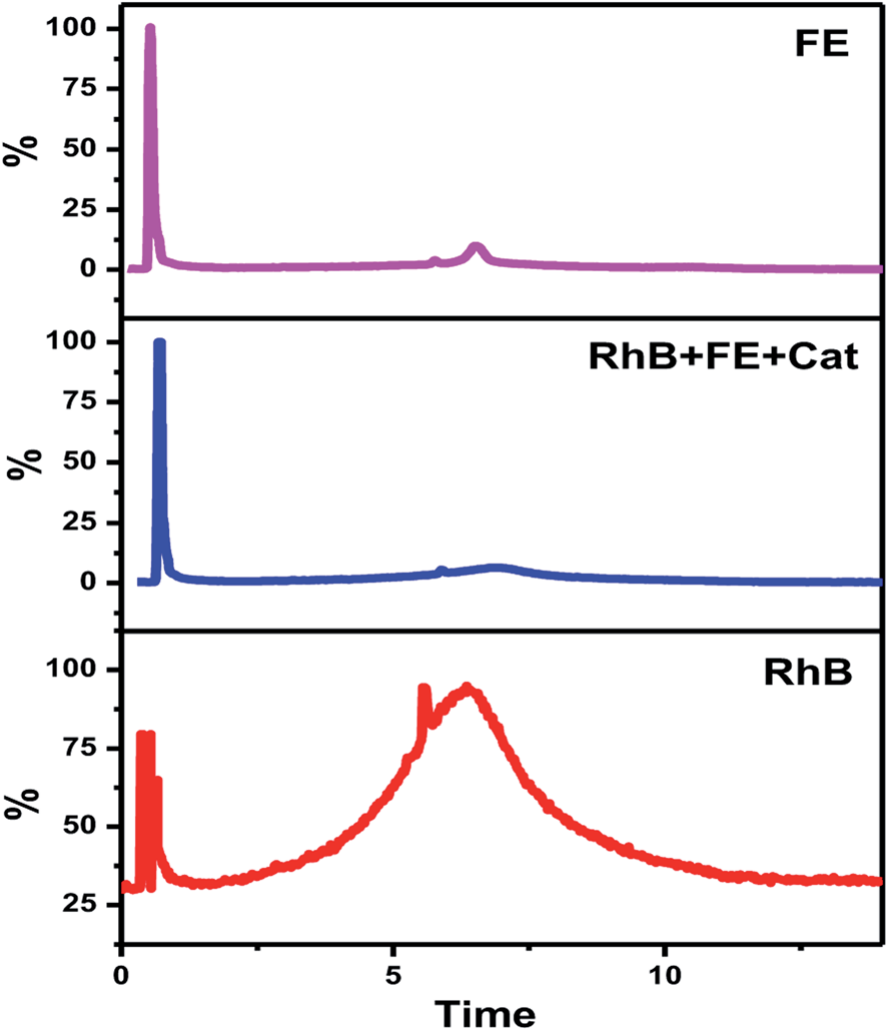
LCMS spectra of the FE, RhB and FE + RhB + Cat.

In this study, the best photocatalyst (Ag-8) was tested for the degradation of fertilizer effluent as well as standard dye with fertilizer effluents. The photocatalyst showed efficiency up to 52% for the fertilizer effluent (FE) in 60 min, whereas standard dye (RhB) with fertilizer effluents (FEs) showed up to 86% photodegradation. This shows that the fertilizer contains remnants of urea and ammonia which are removed up to 52% in the presence of this catalyst under visible light within 60 min. The UV photodegradation plots are shown in Fig. 7a and b separately. Fig. 7a shows the UV absorption peak at 205 nm which can be attributed to urea and its derivatives.^81,82^Fig. 7b shows a low intensity UV absorption peak at 565 due to RhB that was degraded up to ∼86%. Fig. 7c shows the bar diagram of photodegradation efficiency for all sets of samples. Dye in the presence of catalyst shows 96% photodegradation, the FE in the presence of catalyst shows 52% photodegradation and RhB + FE + Cat showed 86% photodegradation. Fig. 7d shows the colour change of the original effluent and dye after the photodegradation experiment. The fertilizer effluent was colourless as evident in the figure but had impurities along with the presence of suspended solids. These executed experiments were then analysed by LCMS spectroscopy and it was inferred that in the standard dye RhB, the concentration of the as prepared dye is very high as shown in Fig. 8 which is close to 99%. This gives the intense broad peak at retention times (RTs) 5.87 and 6.60 min. The FE also had a peak at a RT of 6.83 which can be attributed to trace amounts of urea and ammonia. When these samples were treated with the Cel/Ag_3_PO_4_ photocatalyst (Ag-8), the recorded LCMS spectra showed a decrease in the peak intensity which was found up to 5–10%. This clear evidence demonstrates that the Ag-8 photocatalyst is highly efficient not only for standard dye pollutant but also for the fertilizer effluent sample.

#### 3.10.1 Photodegradation mechanism

Here, RhB was used as a model pollutant to evaluate the degradation efficiencies of cellulose supported silver phosphate nanostructures. The adsorption equilibrium is evaluated up to 30 min in which Ag-0 showed less adsorption ability in the dark whereas, Ag-8 showed higher adsorption ability. The fertilizer effluent mixed with RhB dye also showed positive results for the successful photodegradation using the Ag-8 photocatalyst. When visible light is incident on Ag_3_PO_4_, electrons are generated at CB (0.21 eV) and at VB (2.69 eV). Due to the less band gap width and electrical conductivity, cellulose would serve as an acceptor of photoexcited electrons. Hence electron transfer from Ag-8 to cellulose counterattacks it from combining with Ag-8. The reduction of Ag^+^ to metallic Ag takes place during the photodegradation leading to increased stability. Cel reacts with O_2_ adsorbed on its surface which continually oxidizes the adsorbed dyes by releasing compounds like H_2_O and CO_2._ Photogenerated holes with strong oxidizing power decompose RhB/effluents into inorganic molecules.

## 4. Conclusion

Isolation of cellulose from waste fruit peels was carried out and was further used to design cellulose supported nanostructures. The obtained cellulose from the waste peels showed a 28% higher crystallinity index due to the acid treatment. Cellulose supported silver phosphate nanostructures were successfully developed by an *in situ* approach by altering the amounts of cellulose. All photocatalysts followed pseudo first order kinetics with the maximum observed rate of 0.024 min^−1^ within 60 min leading to 96% photodegradation. Also, the fertilizer effluent (FE) was tested to check the photodegradation efficiency. The RhB dye with Cel/Ag_3_PO_4_ showed 96% degradation efficiency, while the FE and the mixture of FE + RhB showed 52% and 86% degradation efficiency respectively. Disadvantages related to the compound Ag-8 can arise only in rare cases that are concerned with obtaining yield of isolated cellulose. A huge quantity of waste is needed to obtain few grams of cellulose after lyophilisation. If the pre-treatment steps in extracting cellulose are not executed properly, it may create a hindrance in photodegradation experiments which may result in insoluble pectin and hemicelluloses resisting photoactivity. Thus, cellulose acted as an environmentally compatible, economical, and best natural support to be incorporated for improved photostable semiconducting nanostructure formation. By varying the sources of waste for isolating cellulose, one can obtain its different morphologies and sizes which can create composites with multiple semiconducting materials for fabricating novel photocatalysts and treating real samples from industries.

## Conflicts of interest

There is no conflict of interests to declare.

## Supplementary Material

NA-002-D0NA00181C-s001
