# Supplementary material for: Agro-waste extracted cellulose supported silver phosphate nanostructures as a green photocatalyst for improved photodegradation of RhB dye and industrial fertilizer effluents
Source: Nanoscale Adv. 2020 Jun 17;2(7):2870–84. doi: 10.1039/d0na00181c (PMC9417693; doi:10.1039/d0na00181c)
Supplement: NA-002-D0NA00181C-s001 [file NA-002-D0NA00181C-s001.pdf]

Electronic Supplementary Material (ESI) for Nanoscale Advances.
This journal is © The Royal Society of Chemistry 2020

Agro-waste extracted cellulose supported silver phosphate nanostructures as green photocatalyst for improved photodegradation of RhB dye and industrial fertilizer effluent

Neha Tavker[a], Umesh K Gaur[bc]* Manu Sharma[a]*,

[a]School of Nanosciences, Central University of Gujarat, Sector 30, Gandhinagar 382030, India

[b]Department of Physics, National Institute of Technology, Jalandhar, Punjab, India

[c] Institute of Plasma Research, Gandhinagar, Gujarat, India

Supplementary data

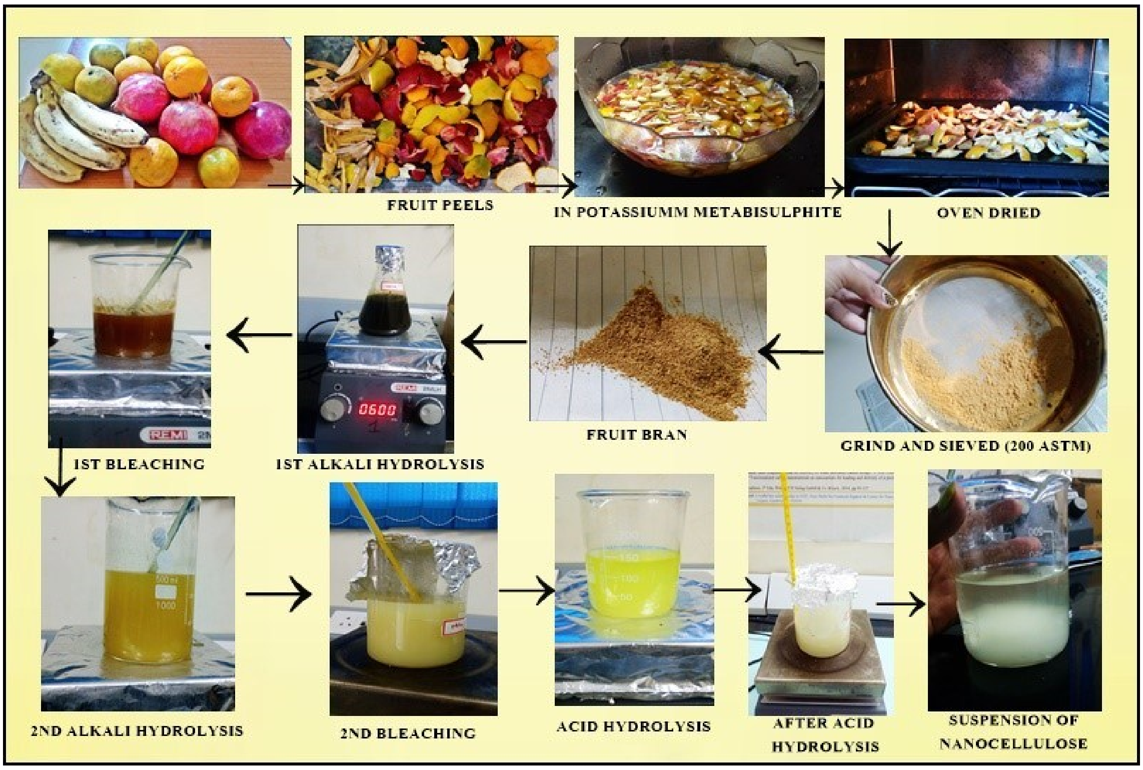

Figure S1: Steps followed for isolation of cellulose (Cel) from waste fruit rinds

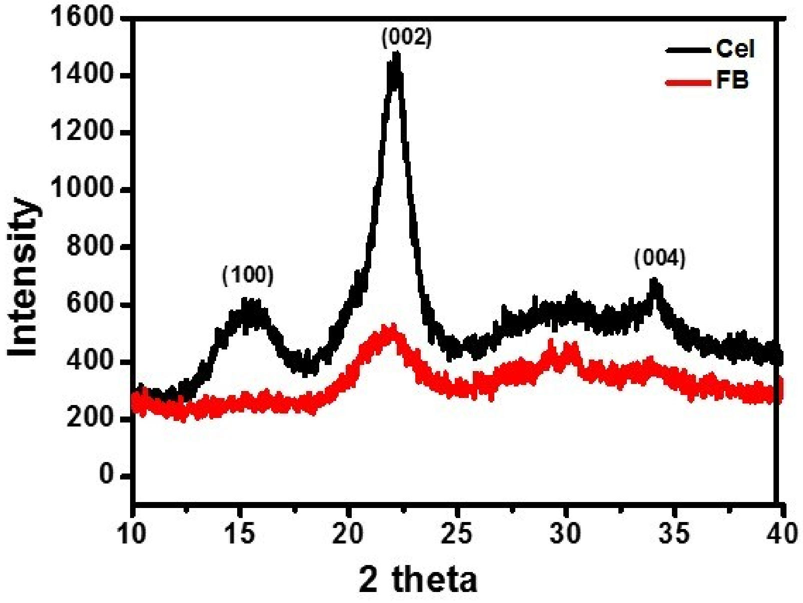

Figure S2: X-ray diffraction pattern of fruit bran (FB) and cellulose (Cel)

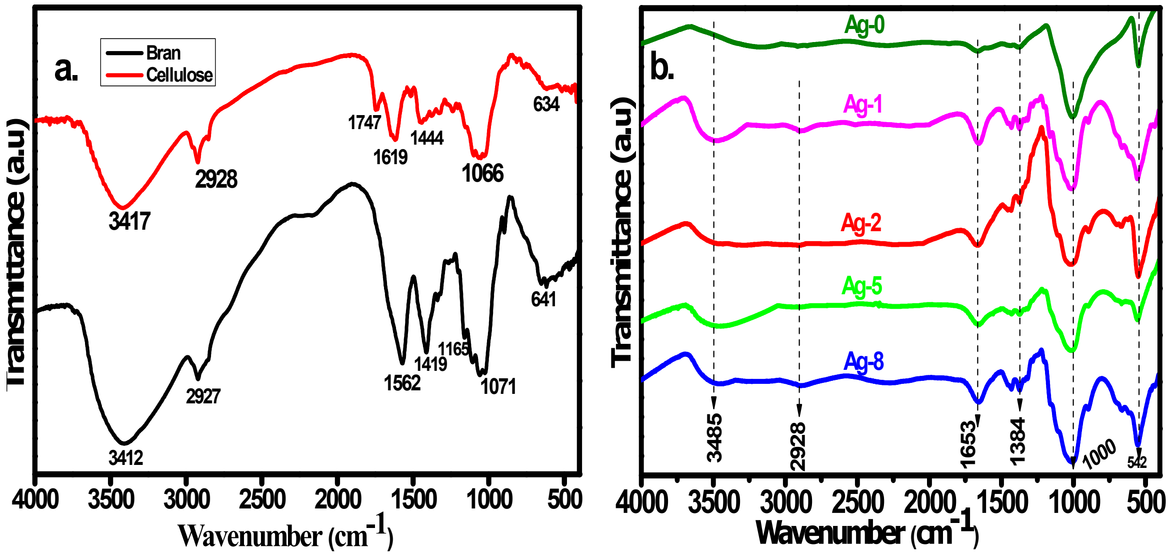

Figure S3: (a) FTIR spectra of fruit bran and cellulose (b) FTIR spectra of cellulose supported Ag_3PO_4 nanostructures [Ag-0 to Ag-8]

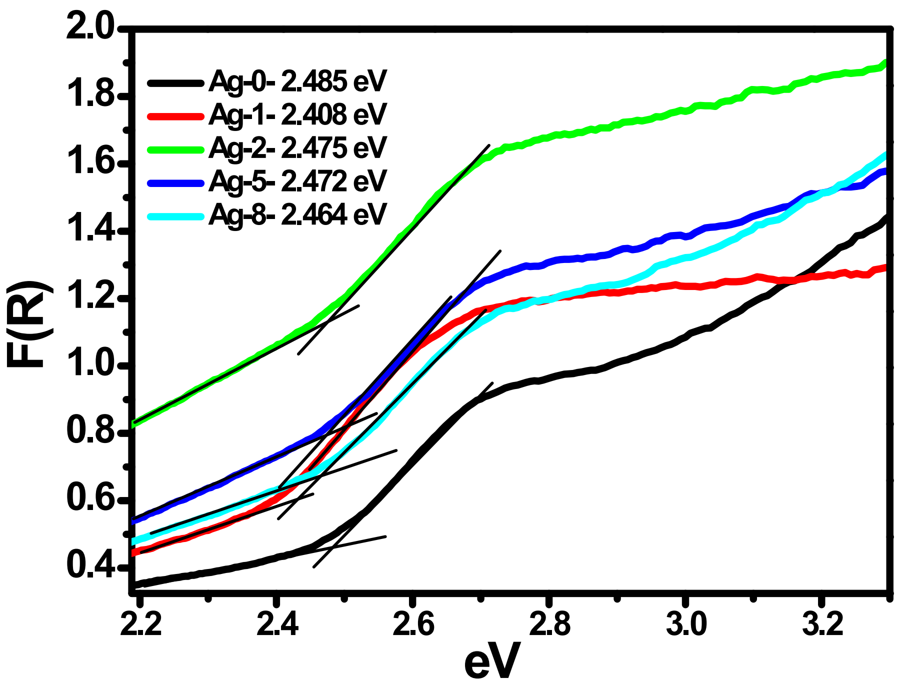

Figure S4: Diffused reflectance spectra of cellulose supported Ag_3PO_4 nanostructures [Ag-0 to Ag-8]

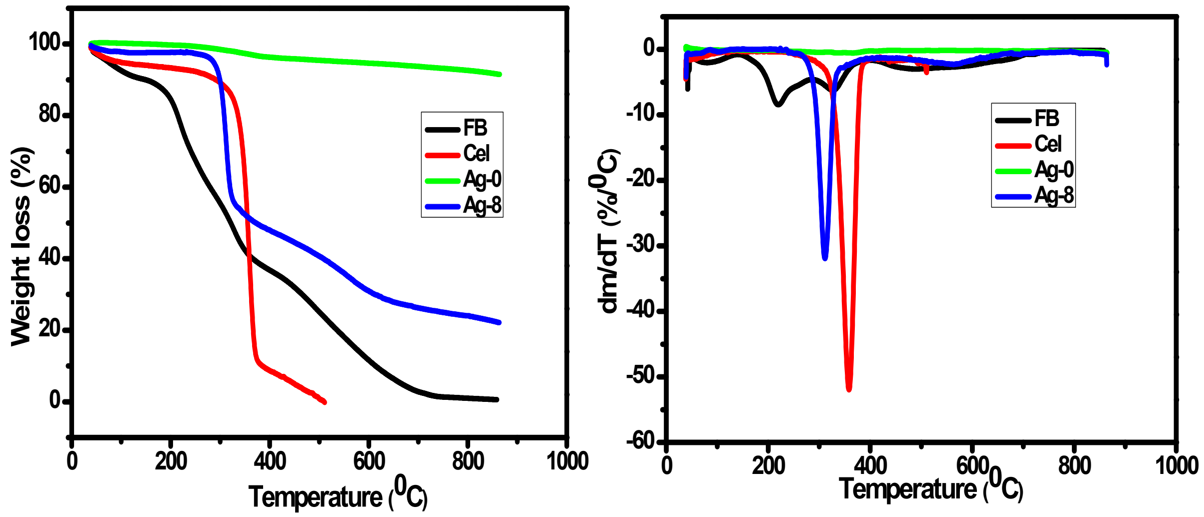

Figure S5: TGA and DTA curves of fruit bran (FB), cellulose (Cel), silver phosphate (Ag-0) and cellulose supported Ag_3PO_4 nanostructures (Ag-8)

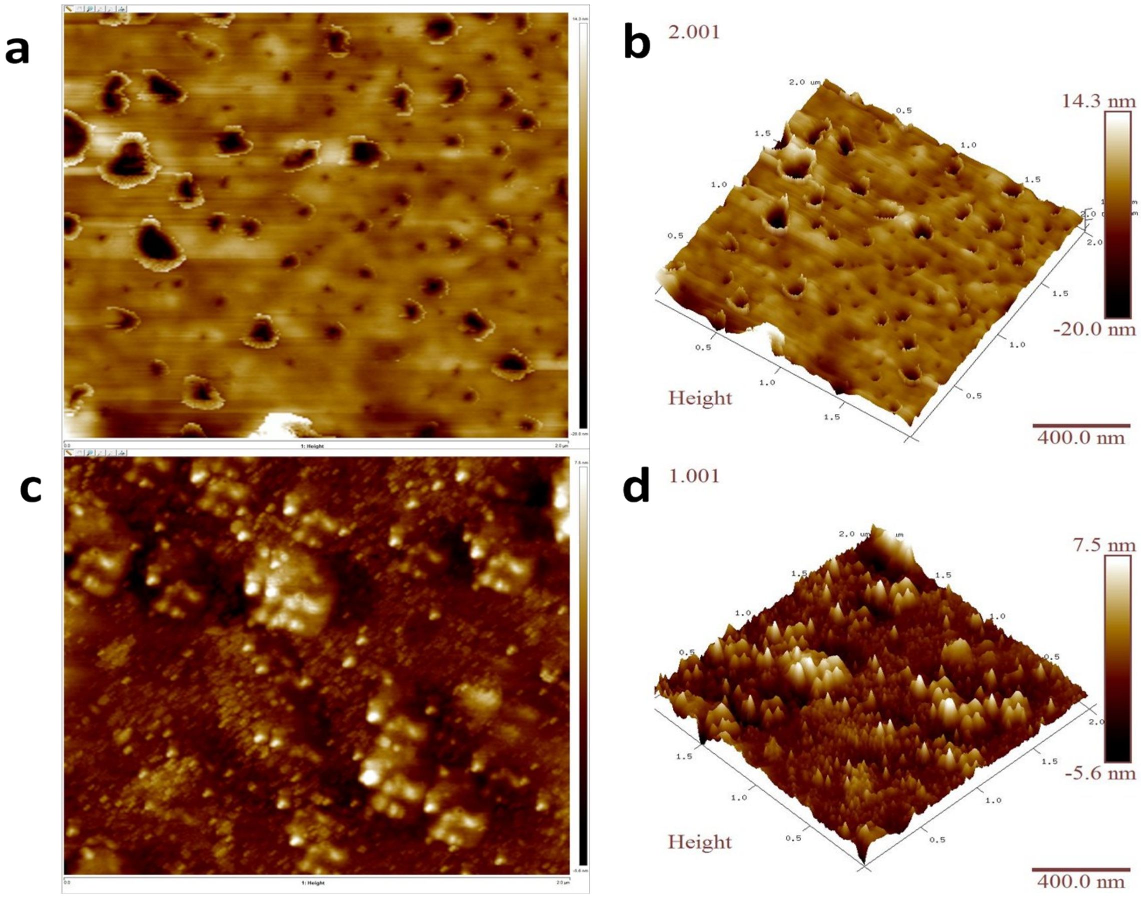
a
b
2.001
14.3 nm
-20.0 nm
Height
400.0 nm
c
d
1.001
7.5 nm
-5.6 nm
Height
400.0 nm

Figure S6: AFM micrographs of a.) Fruit bran (FB) in 2-dimension b.) Fruit bran (FB) in 3-dimension c.) Cel (Cel) in 2-dimension d.) Cel in 3-dimension

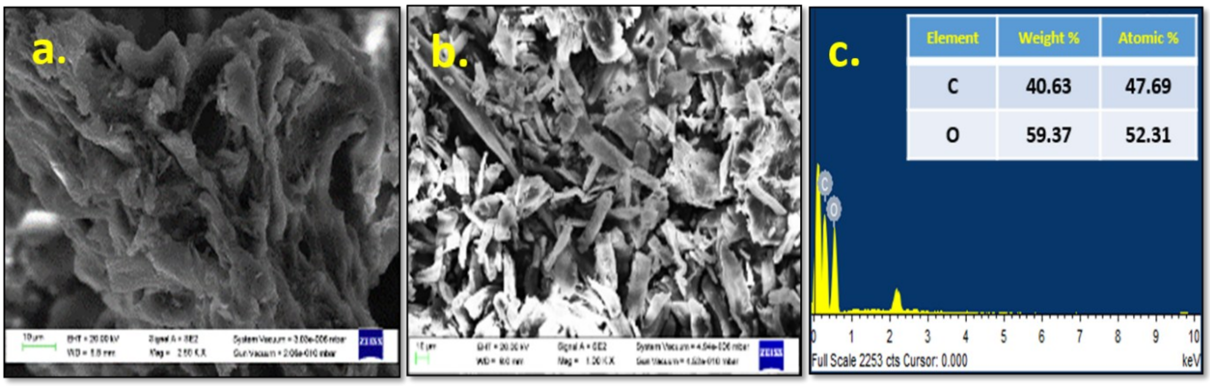

Figure S7: FESEM images of a.) Fruit bran (FB) b.) Cellulose (Cel) c.) EDAX spectra of Cel
